# AGING IN RURAL ETHIOPIA: IMPACT ON FILIAL RESPONSIBILITY AND INTERGENERATIONAL SOLIDARITY

**DOI:** 10.1093/geroni/igz038.549

**Published:** 2019-11-08

**Authors:** Margaret E Adamek, Messay Gebremariam, Abraham Zelalem

**Affiliations:** 1 Indiana University School of Social Work, Indianapolis, Indiana, United States; 2 Addis Ababa University, Addis Ababa, Ethiopia, Ethiopia; 3 Ambo University, Ambo, Ethiopia, Ethiopia

## Abstract

As migration and urbanization continue to progress in developing nations, the filial support and traditional support mechanisms that serve as buffers against the plight of older people are diminishing. Agrarian families find themselves in a rapidly changing world that severely limits their ability to assume caregiving roles for elders. With these trends in mind, a phenomenological approach was used to explore the lived experiences of 10 rural elders in Ethiopia. Prominent themes in the elders’ narratives was the nostalgia of filial responsibility and intergenerational solidarity in the “good old days” and a strong sense of devaluation. Elders expressed feeling devalued by their children, grandchildren, and youth in general. As one elder shared, “These days, there is no respect for an older person. We are treated like a broken utensil thrown away which is considered as useless anymore.” Compared to how they treated their own parents, elders believed that their children’s sense of moral obligation was weak and unreliable. Interactions with children and grandchildren were described as abusive, undermining, and embarrassing, triggering deep sorrow. Elders were pessimistic about the prospect of reliable caregivers, even expressing a wish to die before they become dependent on others for care. Despite the challenges they face in their daily lives, all participants viewed aging as a privilege that should be celebrated. As traditional family support structures in developing nations continue to deteriorate, new models of community-based care are needed to ensure that elders can expect adequate care throughout their lives.

